# Twenty-Five Years of Convoluted Health Reforms in Mexico

**DOI:** 10.1371/journal.pmed.1000124

**Published:** 2009-08-18

**Authors:** Núria Homedes, Antonio Ugalde

**Affiliations:** 1 School of Public Health, University of Texas-Houston, Houston, Texas, United States of America; 2 Department of Sociology, University of Texas-Austin, Austin, Texas, United States of America

## Abstract

Núria Homedes and Antonio Ugalde discuss 25 years of reform to the Mexican health care system and argue that although costs and accessibility have increased, health inequities, efficiency, productivity, and quality of care have not improved.

**Summary Points**

- Mexico's neoliberal health care reforms began in 1983 as a condition for Mexico to receive loans from the World Bank (WB) and International Monetary Fund (IMF), which were needed because of the 1980s world recession.
- The first reform (1983) was a failed attempt to decentralize the Ministry of Health (MoH) by transferring financial responsibilities without devolving adequate decision-making authority to the states.
- The second reform (1994) advanced the decentralization of the MoH and attempted to increase the exposure of the major public social security scheme to private sector competition.
- In 2003, a third reform, the Seguro Popular (SP), emphasized improved access and services for the poor.
- Although accessibility has increased, the Mexican reforms have not resulted in significant reductions of health inequities, or in increased efficiency, productivity, or quality, despite their costs.

## The Socioeconomic and Health Context

Mexico is a large country (population 109 million) with a per capita income of US$8,300 (purchasing power parity US$12,800) in 2007, and as can be seen in Table 1, a highly stratified society [1]. In 2006, Mexico spent about 6.6% of its gross domestic product (GDP) on health care, of which 44% was public expenditure (see Table 1) [2].

**Table 1:** Socioeconomic and health disparities.

Variables	National Average and Range (Lowest and Highest Values)
Education index (2002) [44]	0.82 (state range 0.74–0.90)
Income index (2002) [44]	0.74 (state range 0.59–0.90)
Human development index (2004) [45]	0.81 (state range 0.71–0.88) (municipal range 0.38–0.91)
Households with access to water (2005) [3]	94.5% (state range 85.2–98.4)
Life expectancy (2005) [22]	73 y old for men, 77.9 y old for women (There is a 10-y difference in life expectancy between the poorest and richest groups.)
Infant mortality rate (2004) [45]	19.7 per 1,000 live births (state range 14.4–26.3)
Maternal mortality rate (2005) [22]	63.4 per 100,000 live births (state range 9.6–126.7)
Mortality due to infectious diseases (preventable and avoidable if there is timely access to health care) [3]	In poor communities, 25% of deaths for children <5 y of age are due to infectious diseases; in affluent communities, the corresponding figure is 5%.
Health resources	
Per capita expenditure 2005 [22]	US$498 per capita (state range 316–1,103)
Private health care expenditures 2005 (95% out of pocket) [22]	54% of health expenditure is private (state range 28.5%–76.5%). In 2003 [41] health expenditures represented 8.5% of income for lowest income decile and 2.6% for the highest income decile
Public health expenditure as percent of GDP (2006) [3]	2.9% of GDP (state range 2–8.2%)
Physicians per 1,000 population (2005) [22]	1.9 (state range: 1–4)
Beds per 1,000 population (2005) [22]	1.1 (state range: 0.6–2.5)
Nurses per 1,000 population (2005) [22]	2.2 (state range: 1.3–4.6)

GDP, gross domestic product.

Constitutionally, Mexico is a federation of 31 states and the Federal District, but the federal government has always maintained centralized political and fiscal power. During most of the 20th century Mexico was governed by one authoritarian political party, the Partido Revolucionario Institucional (PRI), which won practically all elections at all levels of government. The health system evolved along the lines of other Latin American countries (see Table 2). Per capita expenditure varied widely, from US$1,100 through the Petróleos Mexicanos (PEMEX) to US$126 through the MoH [3].

**Table 2:** The Mexican health care system (prior to 1984).

Functions	Public Social Security Schemes for Formal Sector Workers and Families	Uninsured	Affluent
Responsibility for services and typical coverage of total population (percent varies each year according to employment conditions)	IMSS for private formal sector employees 40%	MoH 46%	Private insurers 3%
	ISSSTE for government employees 9%		
	SEDENA & SESMAR for armed forces 2%		
	Petróleos Mexicanos (PEMEX) for oil workers less than 0.5%		
Financing	Social Security schemes were financed from three sources: the employer, the government, and the employee. The proportions paid by each source were different for each scheme.	Government (mainly federal with some state contributions)	Private funds
Health care providers	A network of clinics and hospitals staffed and operated by the different schemes	A network of clinics and hospitals staffed and operated by the MoH. Some states and municipalities had developed their own network.	Private network
		The IMSS-COPLAMAR program, which was financed by the MoH and operated by the IMSS, provided health care mainly in rural areas.	
Access to services	Free at point of service (including medications)	Free at point of service (including medicines for priority programs)	Varied
Per capita expenditure	Large variations depending on the type of scheme	Varied by state	Varied

An important early innovation in health care was the extension of free Instituto Mexicano del Seguro Social (IMSS) services to very poor rural areas through a program known as the Coordinación General del Plan Nacional de Zonas Deprimidas y Grupos Marginados (COPLAMAR, General Coordination of the National Plan for Deprived Areas and Marginal Groups). Beginning in 1973 in selected regions, the IMSS-COPLAMAR program significantly broadened access to quality primary and hospital care. Users were highly satisfied with this program. In 1984, the infrastructure and care responsibilities of COPLAMAR were transferred to the 14 states that accepted the decentralization reform (see below), and services deteriorated significantly [4],[5], thereby undermining Mexico's most successful health program servicing underserved communities. The program is still active in the states that did not decentralize and is now called IMSS-Oportunidades.

## The First Health Care Decentralization Reform: 1983–1994

In the early 1980s, the economy of Mexico suffered its worst recession since the Great Depression. The peso was greatly devalued [6], unemployment soared, and real income plummeted. In addition, an oil glut reduced demand for Mexican oil. Oil production is a nationalized industry in Mexico and the country's first source of income. Consequently, the PRI-led government did not have sufficient cash reserves to repay its accumulating national debt. The WB and the IMF were ready to lend but, as a condition of their loan, they demanded, as they had done in other countries, that Mexico reduce its public social expenditure, including its expenditure on health and education [6].

At that time, the WB promoted the decentralization and privatization of health services with the objective of transferring fiscal responsibility to states, municipalities, and users to free the central government's resources and thus expedite the repayment of public debt. However, the labor unions successfully opposed the decentralization and privatization of IMSS, which was temporarily divided into eight administrative regions, and the first reform was, therefore, limited to the decentralization of the MoH [7]. Following the guidance of the WB, the MoH presented the reform as a means to improve efficiency and quality, increase productivity, and make the health care system more participatory.

Decentralization agreements specified the new responsibilities of individual states, including the requirement for a substantial increase in funding from the state. Depending on the economic condition of each state, between 20% and 40% of health expenditure became state mandated. The decentralization was expected to be completed by 1986, but by 1987 only 14 of the 31 states had decentralized (see Table 3).

**Table 3:** Organization of health care for the uninsured in the 14 decentralized states (1984–1994).

Responsibility for Services	State Health System
Financing	Federal government, state governments, and user fees. States committed to increase their allocations to health,
Health care providers	A network of state health services: all public facilities to be managed by the state health secretariats (including IMSS-COPLAMAR). Federal health employees refused to become state employees because salaries and fringe benefits tended to be lower. Labor unions refused to accept the decentralization. All state health workers were given the opportunity of becoming federal employees. Ironically, most workers at the state health secretariats are now federal employees
Access to services	User fees for services and medicines. Medicines for priority programs were free.
Per capita expenditure	Varied by state
Devolution of decision-making power	Minimal: programs continued to be designed by MoH; states had very little control over financial resources (except for user fees). Personnel appointments continued to be made by MoH

Most studies [8]–[12] have concluded that this first decentralization effort, which ended with the change of government in 1988, failed to improve efficiency, increased health inequities, and had a negative impact on quality. The official estimate of the cost of this first reform is staggering: 140 billion pesos [13], or approximately US$452 million.

The 1988–1994 administration (which was also led by the PRI), reversed course and stalled the decentralization of health services. The president's office managed large new social services programs and funded local groups of its choice—a shift that was perceived by observers as an attempt to recentralize decision making. State and municipal PRI politicians felt bypassed and were outraged, citizens grew impatient at the lack of services, and popular support for the PRI reached an historical low. The health service delivery model continued as in the previous administration.

## The Second Health Care Decentralization Reform: 1994 Onwards

The PRI government elected in 1994 understood that to win future elections it would need to modernize by increasing public participation and decreasing its traditional authoritarianism. It quickly launched a program known as The New Federalism. Decentralization was one of this program's key components. The central government increased health funding to the states and transferred decision-making power (see Table 4). By 1999, all the states and the Federal District had signed the new decentralization agreements [14].

**Table 4:** Organization of health care for the uninsured after the second decentralization reform (1996).

Responsibility for Services	State Health System
Financing	Federal government, state governments, and user fees
Health care providers	A network of state health services: all public facilities to be managed by the state health secretary. The majority of state employees became federal employees, states gained some control over human resources, programs, and finances
Access to services	User fees for services and medicines. Medicines for priority programs were free. Attending physicians often waived fees for the indigent.
Per capita expenditure	Varied by state
Devolution of decision-making power	States obtained some control of personnel. In coordination with the state branch of the worker's union, they could transfer and fire personnel, and recommend new federal hires.
	MoH transferred ownership of physical infrastructure to the states.
	The states were allowed ample discretionary power to spend federal transferred funds, except the funds allocated to human resources, although they were able to use the unspent personnel funds (due to absenteeism, leaves of absences) at their discretion.

Central fund allocations to the states continued to be based on historical budgets. In particular, the powerful and wealthy states did not want to see their allocations reduced in order to increase the allocations of poor states. Indeed, funding disparities persisted and even increased as the wealthy states increased their own allocations and were able to charge higher copayments. Case studies carried out in several states found that decentralization increased health coverage (through an increase in the number of health facilities and the use of mobile teams), but that the health care system remained inefficient, with relatively low levels of productivity in spite of new incentives. Community participation also remained very weak [14].

## Failed Attempts at Health Care Privatization

Throughout these years, the WB continued to promote the privatization of health services in response to a policy that promoted market competition as a way to increase productivity and quality. In 1985 the Mexican minister of health founded the private Health Foundation (Funsalud), which was funded by transnational corporations operating in Mexico including tobacco, pharmaceutical, and food corporations (http://www.funsalud.org.mx/).

The WB worked closely with Funsalud, which was led by Julio Frenk, and with Juan Luis Londoño, the Colombian minister responsible for the 1993 Colombian health reform. In 1997 Frenk and Londoño coauthored an article entitled “Structural pluralism: towards an innovative model for health system reform in Latin America” that outlined the Colombian and WB's health reform policy tenets [15]. These tenets stated that: (1) publicly run systems are inefficient and of poor quality; health care based on market competition can achieve higher productivity, efficiency, and quality; and (2) market competition creates a more flexible labor force.

In the structured pluralism model for health care, the funding follows the patient, whereas in the traditional model funds are allocated to health care networks regardless of the services provided [15]. Structured pluralism advocates universal coverage through insurance-based systems in which the premium for the poorest people is subsidized by the government and in which public and private institutions compete to capture clients. Government regulates the system by monitoring performance and financing the services for the poor.

In 1997, the WB granted a loan of more than US$700 million to Mexico's health sector, but the terms and conditions of this loan were negotiated without the knowledge of the National Congress. A leak to the press by Congressman Rojas Arreola, a member of the Partido de la Revolución Democrática (PRD) center-left opposition party, revealed that one of the components of the loan potentially weakened IMSS by allowing its beneficiaries to choose between available public and private providers [16]. Rojas Arreola and the labor unions interpreted these changes as the beginning of the privatization of IMSS, and, with the support of Congress, derailed the attempt.

However, the formula used to finance IMSS was changed by a law approved in 1995, which became effective in 1997. The government's contribution was increased and the employees' contribution was decreased, especially for those with higher incomes. These changes and the country's economic downturn decreased the financial and human resources of IMSS [17]. As a result, services deteriorated and some felt that major structural changes were needed, including an increase in the role of private insurers and private providers. Interestingly, a 2005 article by Frenk and Knaul indicates that Mexico intended to increase the role of the private sector: “The [Mexican] reform builds on earlier and ongoing experiences in other Latin American countries such as Colombia and Chile…” [18].

## People's Health Insurance (Seguro Popular)

In 2000, after almost 70 years in power, the PRI lost the national elections to the Partido de Acción Nacional (PAN), a conservative party, and Frenk was appointed minister of health. Aware that the labor unions would block the privatization of IMSS, a third health care reform was proposed called the System for Social Protection for Health (SSPH). This reform, which is commonly known as the People's Health Insurance or Seguro Popular (SP), was approved by a Congress controlled by opposing parties.

SP is a voluntary family health insurance program for the uninsured that was designed by the federal government and financed through conditional grants offered to the states by the federal government for its implementation. It was promoted as a program to provide free insurance to the poorest of the poor and contained a firm commitment that services would be publicly provided [17]. Notwithstanding this commitment, the MoH has allowed SP to contract with the private sector for health services. The roll-out of SP began in 2004, diverting focus from the previous decentralization effort.

As indicated earlier, by 2000 decentralization was making some headway although there were problems. A knock-on effect of this ongoing decentralization was that when the MoH found that the states implementing SP were not always following the intended design, decentralized decision making prevented the MoH from intervening. Indeed, the minister viewed decentralization as an obstacle to SP [19].

The organization of health services for the uninsured after the third reform is presented in Table 5. Family contributions to SP are based on a sliding-fee scale, and are waived for families in the lowest two income deciles and for those in the third lowest income decile with a child under 5 y of age. The federal contributions are based on a per-enrolled family fee plus a solidarity supplement for the poorer states. These states have the greatest proportion of poor and uninsured. Because the state contributions to the program are set on a per-enrolled family basis, the poor states that have the highest proportion of poor and uninsured have to make a higher contribution than the wealthier states, thereby increasing geographical inequity.

**Table 5:** Health care delivery for the uninsured after the creation of SP.

Functions	Uninsured Not Affiliated to SP (Remains Basically Unchanged)	Affiliated to SP
Responsibility for services	State health system	State Health System. The System for Social Protection for Health (SSPH), also referred to as SP, decides the services to be provided to the insured, and the protocols to be followed
Financing	Federal government, state governments, and user fees	The financing formula is very complicated. The MoH and the states make a fixed per family contribution. Enrolled families contribute to the system based on a sliding-fee scale. The federal government allocates extra funds to the most marginalized states.
		Family premiums are waived for families in the lowest two income deciles and for those in the third lowest income decile with a child under 5 y.
Health care providers	A network of state health services: all public facilities to be managed by the state health secretary.	States can decide, usually a network of private and public facilities and providers.
	The majority of state employees are federal employees; states have some control over human resources, programs, and finances	Often the state is unable to provide mandatory package of services and there is a need to contract with the private sector.
Access to services	User fees for services and medicines.	Free at point of services (includes 312 medicines)
	Medicines for priority programs were free. Attending physicians often waived fees for the indigent.	
Per capita expenditure	Varied by state	Varies by state, but it is higher than for people unincorporated to SP who remain uninsured.

SP beneficiaries receive a package, which is periodically increased, of free health services, pharmaceuticals, and care for some catastrophic events. As of December 2008, the package covered 266 interventions, 18 catastrophic events, and 312 medicines [20], and could be provided by accredited public or private clinics and hospitals. Nineteen states and the Federal District have contracted with the public and private sector and 11 states have contracted with institutes of social security for the provision of services. Michoacan is the only state that has not contracted out any services. Care for most catastrophic events (84%) is privately provided [21].

Despite the public sector having low productivity levels (in 2005 general practitioners and specialists had an average of 18 and 2.4 consultations per day, respectively) and relatively low hospital occupancy rates (72.2%) [22], SP has required heavy infrastructure investments and substantial recruitment of personnel for its implementation including: 1,724 new health units [10] and 102,000 additional workers with temporary contracts, i.e., a flexible labor force [23]. According to a source in the Mexican Ministry of Health, who wishes to remain anonymous, in August 2008 about 45,000 of these temporary workers were to receive regular staff status, thus further increasing the cost of SP. According to 28 states these resources are not sufficient [21]. The cost of the SP program for the year 2007 was US$2.75 billion or an average of US$377 per family (with the federal government responsible for 69% of the cost).

### Assessments of SP

The objectives for the creation of SP are presented in Box 1. Several recent reports have assessed the SP. Positive assessments of SP by Frenk and colleagues that were published in *The Lancet* in 2006 and in an earlier publication by Knaul and Frenk [18],[24]–[28] have been questioned by independent researchers. Specifically, concerns exist regarding cost effectiveness and impact on equity of SP [17],[29]–[31]. For example, by the end of 2007, the SP had enrolled mainly people who did not have to pay and about 35 million persons (61.9% of the eligible population) remained uninsured.

**Box 1. SP Objectives [18],[24]**

- Establish a system of universal access based on social insurance
- Improve the allocation of resources by defining a package of cost-effective interventions
- Decrease out-of-pocket expenditures, especially for the poor
- Make the distribution of federal resources to the states more equitable
- Increase competition among service providers to raise productivity levels and improve the quality and efficiency of the health sector
- Protect the funding of public health interventions
- Protect families from excessive health expenditures

According to early evaluations [21],[32] the SP has improved access to medical care including the treatment of chronic diseases (diabetes, asthma, arthritis, and high blood pressure) and the provision of pharmaceuticals and dental care. SP has also lowered out-of-pocket expenditures for the enrollees (see Box 2). A more recent study [33] has confirmed that SP has successfully reduced catastrophic and out-of-pocket expenditures, especially among the poorest, even though it has had no effect on medication spending, health services utilization, or health outcomes.

**Box 2. Independent Assessments of SP's Impact**

- About 62% of SP enrollees were able to access needed services compared to 54% of the unenrolled [32]
- During the 2 wk prior to one survey, free medicine was received by 68% of SP enrollees and by only 60% of those not enrolled [21]
- No differences were found between enrolled and unenrolled people in access to preventive services [21]
- SP enrollees tended to access health services less frequently than the rest of the population [43]
- Two surveys reported a difference of 2.2% to 3.2% in enrolled families and unenrolled families seeking coverage for catastrophic events in the SP program, respectively, but a third survey failed to document any difference [21]
- Two surveys reported 17% and 18% lower out-of-pocket health expenditures among the families enrolled in SP compared to unenrolled families, respectively; a third survey did not find a significant difference [21]
- Unfortunately no information is available on the quality and efficiency of the services provided through the SP system. Urbina commented that the quality of the services is perceived as deficient, which may have a negative impact on the re-enrollment of eligible families [32]

However, some critics suggest that the SP was designed to allow the private sector to provide care and to compete with public providers thereby diminishing the public sector role [17]. In other words, the implementation of SP in Mexico has followed the examples set by Chile [34] and Colombia [35] in the reform of their health care services.

### Poor Coordination and Implementation Constraints of SP

The administration of SP is complex. At both the federal and state levels, there are several departments and units involved in the implementation of the SP, and, unfortunately, coordination among them has been poor. Furthermore, since its creation, SP has evolved without a detailed long-term view of the system and without appropriate management and evaluation tools [32]. The expansion of entitlements and changes to entitlements that have occurred as the federal government has understandably increased subsidies have generated confusion among providers and beneficiaries, and continue to threaten the appropriate implementation of the program. As Sojo [7] and Urbina [32] have pointed out, the designers of the reform did not foresee the outcomes of certain policy directions.

Boxes 3 and 4 list causes of administrative dysfunction in SP and documented implementation problems and illustrate additional potential implementation constraints.

**Box 3. Causes of Administrative Dysfunction within SP [21]**

- Decision making is dispersed among many divisions and units of the federal MoH and state health secretariats
- Decentralized states do not always feel obligated to follow the directives of the federal MoH
- There are discrepancies among the federal and state laws and regulations
- Many federal and state employees are still unfamiliar with the regulatory SP framework
- There has been a failure to establish a monitoring and evaluation system to control the performance of the SP at the local and state levels
- The states have limited managerial capacity

**Box 4. Documented SP Implementation Problems [21],[39]**

- Inadequate guidelines for the accreditation of SP facilities
- Federal government funding below promised levels
- The use of funds for purposes other than those for which they were intended; for example, funds allocated for catastrophic events have been used to cover immunization programs, and funds earmarked by the MoH for health needs are being used by the states for nonhealth purposes
- Bureaucratic rigidity and slow implementation of contracts
- Inaccuracies in the data gathered by the information systems (including financial reports) and deficiencies in the processes used to determine the income of SP eligible families
- Tensions between the state ministries of health and the person responsible for the financial management of the SP
- Limited progress in signing SP portability agreements among states

### Efficiency of SP

Some of the implementation shortcomings of SP presented in Boxes 3 and 4 create inefficiencies in health care provision. For example, the small amount of funds collected through family fees may not offset the cost of collection. Also, the determination of eligibility for fee waivers is costly and is done annually.

Critics note that SP has further fragmented and stratified Mexico's health system [30],[36],[37], a process that is likely to have a deleterious effect on its efficiency. Moreover, because public (health care) employees can earn additional income by working in the private sector, with the implementation of SP they have even more incentives to decrease their productivity in the public sector and to divert patients to their private offices. These conflicts of interest are expected to grow as the role of the private sector expands.

In addition, the undisclosed increase in administrative costs associated with the implementation of SP is worrisome [7], especially when taking into consideration that prior to the SP the Mexican health system had the highest administrative cost of the 30 countries that are members of the Organization for Economic Co-operation and Development [38]. In 2007, Mexico's auditor general confirmed that the SP budget was insufficient to cover all services promised and affirmed that the allocation of hundreds of millions of dollars had not been documented [39], a statement that raises questions about the sustainability of the SP, especially in view of the current global economic crisis.

### Equity of SP

In 2007 large interstate differences existed in the proportion of persons enrolled in the SP. Some states had enrolled more than 70% of eligible poor families, whereas in other states less than 30% of eligible families had been enrolled [32]. Poorer states and those with the largest number of indigenous persons were the slowest to enroll eligible families [21], probably because, among other reasons, they may not have had the required matching funds. Furthermore, there are unexplained differences in per-family health expenditure by state. In 2007 the average per-family total expenditure was US$377 but varied between US$208 and US$511 between states. Similarly, the average per-family federal allocation to the state was US$259, with a range of US$123 to US$378 across the states [32]. The fact that some wealthier states are receiving higher than average per-family federal allocations while some poor states receive lower than average allocations suggests that an attempt by the federal government to rebalance geographical health inequities has not worked.

## Discussion

Mexico's attempts at health reform have been extremely convoluted. The first decentralization reform of its health care system was a response to requests from the WB and the IMF to free central funds to pay its external public debt during a world recession. After 6 y, only 14 states had agreed to be decentralized. The remaining states understood that decentralization was not accompanied with the additional funding needed to undertake the new responsibilities that decentralization was transferring. The decentralized states soon protested the minimal transfer of decision-making power as well as the demands made by the central government for additional state funds.

The next administration (1988–1994) understood that the cost of decentralization had been high and its achievements negligible, and the decentralization process was halted. This administration took a different position on how to develop the country politically and economically and decided to centralize all new social service programs in the president's office. Politically, the results were catastrophic and political analysts forecasted the end of the PRI hegemony.

Recognizing that political changes were needed to remain in power, the subsequent PRI administration decided to reduce political authoritarianism. One measure it took was to transfer decision-making power and funding to the states, thus initiating the second decentralization of the health sector. There was also an attempt to implement a reform along the lines of the structured pluralism model, which was derailed by the IMSS labor unions.

Finally, new efforts to privatize the delivery of care commenced when SP was launched by PAN at the start of this century. Unfortunately, the designers of the SP ignored the implementation constraints of a program prepared without concurrence from the states. In particular, the decentralized states did not have to adhere to the requisites mandated by the MoH. The ministry soon realized that decentralization was impeding the implementation of SP, and although government documents continued to mention decentralization since the inception of SP, no attempts have been made to advance decentralization further.

One state policy maker in Sonora explained the designers' failure to foresee implementation problems when he referred to the designers: “[as researchers] experimenting with models, always generating ideas that were not very practical… did not have their feet in the real world… kids from Harvard with no social experience, out of touch with the people with needs” [40, p. 192]. Consequently, only a few states had the planning resources to design an insurance scheme for the poor, and now Mexico has 32 variations of the SP, with unforeseeable equity and portability problems.

There are also design incongruities within the SP. It is a voluntary program that promises to have the entire population insured by 2010. Five years after its inception, however, less than 1% of eligible families pay premiums, and 74% of the premiums that are collected come from the state of Tabasco; in all other states practically all the enrollees have had their fees waived. According to the SP designers, the 35 million people who are unenrolled but eligible (most of whom will have to pay premiums) should be enrolled by 2010. However, this group may prefer to continue paying copayments at state facilities unless they perceive a drastic deterioration of quality in these services. This deterioration could occur if the MoH reduces the allocation of resources to the state health services. If such a policy takes place, the 17-y decentralization effort to strengthen the states' health systems would be lost. Families could also decide to join the IMSS program for the uninsured, an option that has been available for a number of years but that very few have chosen, probably because the concept of insurance and prepayment of premiums are not part of the culture among less affluent people.

Although SP must improve in many areas to reach its goals, its designers highlight successful aspects of the program and cite the millions of people enrolled in the program. These successful aspects are inevitable; poor families are insured at no cost as a result of the massive allocation of additional resources that has created two parallel state-run health care systems for the uninsured. Increasing health funding in Mexico is important, but independent evaluations suggest that SP is not the most successful model to achieve equity, efficiency, and quality care.

SP enrollees have always had access to public health services with a copayment. Exempting the poor from copayments might have been a less expensive way to accomplish the same end result as the implementation of SP. Better and more equitable results, probably at a lower cost, might also have been achieved by helping the IMSS to increase its efficiency and workforce productivity, and by expanding its programs for the uninsured. Instead, according to observers, SP may reduce employers' incentives to offer Social Security coverage [7],[37],[41], and SP may expand at the expense of IMSS [18], especially now that the MoH's priority is the SP [42].

Given the lack of continuity for social programs in the past, it is hard to foresee the future for SP with any degree of certainty. Future governments could decide to transfer SP to IMSS or to state health departments to promote a more complete decentralization. Alternatively, they might continue to provide subsidies for those already enrolled in the SP. If the SP program continues to build on the principles of the Colombian reform, however, the effects of SP on the Mexican health care system may not be necessarily positive.
